# Editorial: *Neurophotonics* for you

**DOI:** 10.1117/1.NPh.8.1.010101

**Published:** 2021-03-05

**Authors:** Anna Devor

## Abstract

Neurophotonics Editor in Chief Anna Devor outlines a vision for the journal.

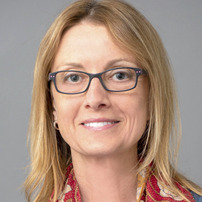



Dear Friend,

With this issue I formally begin my tenure as the editor of *Neurophotonics* with the goal to make it your preferred journal. I assume this role with a strong sense of responsibility and deep appreciation and gratitude to the founding editor Dr. David Boas, all those who serve the journal, the SPIE publications team, and our past and present members of the editorial board.

*Neurophotonics* was launched in 2014 coinciding with the launch of the BRAIN Initiative focused on the development of technologies for advancement of neuroscience. For the last six years, *Neurophotonics’* agenda has been well aligned with this focus on neurotechnologies featuring new optical methods and tools applicable to brain studies. Nowadays, the BRAIN Initiative 2.0 is pivoting towards applications of these novel tools in the quest to understand the brain. At *Neurophotonics*, we see this as an opportunity for growth and expansion. While the focus on optical methods remains at the heart of our agenda, we welcome research that applies these methods to address specific neuroscience hypotheses and questions. Our interest in neuroscience applications spans across the spatiotemporal scales, species, and includes basic research as well as understanding of brain disease.

We have ambitious plans for further improving the quality of the journal to be operationalized through publication of the best science, promoting the interdisciplinary neurophotonics community, dissemination of neurophotonics technologies and experimental data, serving as an educational resource for neurophotonics students, and more. We are happy to introduce new paper types: *Primers*, *Protocols* and *Data Papers*. *Primers* will provide a conceptual overview of a current or emerging optical tool or technology. Our *Primers* will cover basic technical background, current applications, and an outlook on the future impact of these tools and technologies. *Protocols* will describe an original method (or its latest version) that the authors used to generate results published in a peer-reviewed journal. *Data Papers* will describe research datasets. These datasets would have to be deposited in a public repository and made publicly available upon publication.

We look forward to expanding our editorial board and welcoming newly appointed members. We will make all efforts to further improve our peer review to be conducted in a fair and swift manner. We would like to thank our reviewers who have provided thoughtful, critical and supportive feedback to our authors in the past and hope that they will continue to do so in the future. It takes a village to raise a journal, and our goal is to make *Neurophotonics* the one you enjoy reading and find the most useful, illuminating, and informative.

Finally, as am writing these words, the COVID-19 pandemic has claimed over 500,000 lives in the US alone, and many more around the world. This is an unimaginable suffering and loss of human life that has affected all of us. Despite this ongoing struggle, academic research and exchange of knowledge and ideas remain strong. At *Neurophotonics* we see ourselves not only as a publishing platform but as a diverse and vibrant international community that includes our authors, reviewers and readers. At this challenging time, our message to those of you who find themselves deprived and isolated is: you are not alone! You are a part of our community. Stay driven, stay creative, believe in yourself and support those around you. We will overcome this challenge and emerge on the other side of this calamity stronger than ever.

If you have thoughts about specific formats and rubrics that you would like to see in our journal, please let us know by emailing neurophotonics@spie.org. We look forward to hearing from you and invite you to send your best work to *Neurophotonics*!

